# Evaluation of micro Electroretinograms Recorded with Multiple Electrode Array to Assess Focal Retinal Function

**DOI:** 10.1038/srep30719

**Published:** 2016-08-02

**Authors:** Momo Fujii, Genshiro A. Sunagawa, Mineo Kondo, Masayo Takahashi, Michiko Mandai

**Affiliations:** 1 Laboratory for Retinal Regeneration, RIKEN Center for Developmental Biology, Kobe, 650-0047, Japan; 2 Department of Ophthalmology, Mie University Graduate School of Medicine, 2-174, Edobashi, Tsu, Mie, 514-8507, Japan

## Abstract

Full-field electroretinograms (ERGs) are used to objectively assess the mass function of the retina, whereas focal ERGs are used to evaluate the focal retinal function. The purpose of this study was to determine the usefulness of a multiple electrode array (MEA) system for recording *ex vivo* micro ERGs (mERGs) together with multiunit spike responses of the retinal ganglion cells (RGCs) to assess focal retinal function in isolated mouse retinas. The a- and b-waves of the full-field ERGs were present in the mERG. The b-wave was blocked by L-AP4, an inhibitor of the mGluR6 receptor, and the OFF-component was blocked by exposure to PDA, an antagonist of ionotropic glutamate receptors, with a corresponding RGC responses. mERGs were also recorded from mice with progressive retinal degeneration, the C57BL/6J-*Pde6b*^*rd1-2J*^/J (*rd1*) mice, from which conventional full-field ERGs are non-recordable. A blockade of the glutamate receptors indicated that the negative wave of *rd1* mice do not originate from the photoreceptors but from the second or third order neurons. This technique of recording mERGs will be useful in assessing the focal properties of the retinas obtained from eyes with pathology and also to follow the recovery of the physiology of the retina in regenerative studies.

Full-field electroretinograms (ERGs) are used to assess and diagnose retinochoroidal diseases in patients, and they are also used in animal experiments to determine which retinal neurons contribute to the ERGs^1^. Both *in vivo* and *in vitro* methods have been used to record ERGs in animals to examine the physiological properties of the retina. The full-field ERGs represent the changes in the mass retinal field potentials, and the ERG is composed of the sum of the electrical potential changes of the different retinal cell types^2^,^3^. The first component of the full-field ERGs is the negative a-wave which originates predominantly from the rod photoreceptors but also receives some input from the cells of the inner retina that contribute to the OFF pathways. The a-wave is followed by a positive b-wave which originates from bipolar cells and Müller glia cells. The slower c-wave originates from a combination of a slow positive potential from the retinal pigment epithelial cells and a slow negative potential from retinal Müller glia cells in response to a light-induced change in K^+^ in the subretinal space^3^. ERGs can be used to determine which neural elements of the retina are the cause of a specific pathologic condition, and the findings can contribute to making a diagnosis. This has been done in eyes with photoreceptor degenerative diseases and bipolar cell dysfunction.

Local changes are important in studying degenerative processes or in following the recovery of retinal function in regenerative studies, but these are often masked by the mass responses in full-field ERGs. In the clinic and some large-sized animals, focal ERGs and multifocal ERGs (mfERG) are used to evaluate the local retinal physiology. In humans, focal ERGs are used to evaluate the cone photoreceptor function and are performed using background light to suppress the rod responses and to eliminate the effect of stray light. The responses are generally small, and many ERGs are summed to improve the signal-to-noise ratio. A study has been published which reported on the mfERGs recorded from mice under dark-adapted conditions^4^. However, because of the difficulty in focusing the stimuli on focal areas in small eyes *in situ* and in eliminating the effects of stray light, recording mfERGs from mice and rats are still challenging.

A multiple electrode array (MEA) system is an effective *in vitro* method of recording and studying focal neuronal networks and has been used in many research fields^5^,^6^,^7^,^8^. In the retina, this system has been used to assess the spontaneous and light-evoked retinal ganglion cell (RGC) activities^9^,^10^,^11^,^12^,^13^,^14^,^15^. The results of an earlier study^16^ and our study^17^ demonstrated that an MEA can also be used to record focal ERGs from isolated mouse retinas. We showed that this method was more sensitive than full-field ERGs in detecting the acute alterations of the ERGs induced by methyl–*N*-nitrosourea in a mouse retinal degeneration model^17^.

The purpose of this study was to determine whether the mERGs recorded with the MEA system have the a- and b-waves with shapes and properties similar to the a- and b-waves recorded in the full-field ERGs, since previous mERG recordings, including ours, did not clearly demonstrate the b-waves using MEA^17^,^18^. In addition, we also determined the usefulness of the MEA system in assessing the physiological properties of retinas of mice with progressive photoreceptor degeneration, the *rd1* mice. In *rd1* mice, the rod photoreceptors begin to degenerate at about postnatal day 8, and the photoreceptors are mostly lost by 4 weeks after birth. Because the degeneration process is so rapid, it has been reported that it is not possible to obtain full-field ERGs from these mice^19^. We studied mERGs during the degenerating process in these mice and studied the different components of the mERGs with the use of two glutamate receptor inhibitors. We were able to record light-evoked mERGs together with consistent RGC spike responses. However, the negative wave did not originate from the photoreceptors as normally observed in wild type retina, but from the OFF bipolar cells and/or third order neurons.

## Results

### Reproducible recordings of a- and b-waves of mERGs in reference to RGC multiunit responses

**Figure 1:**
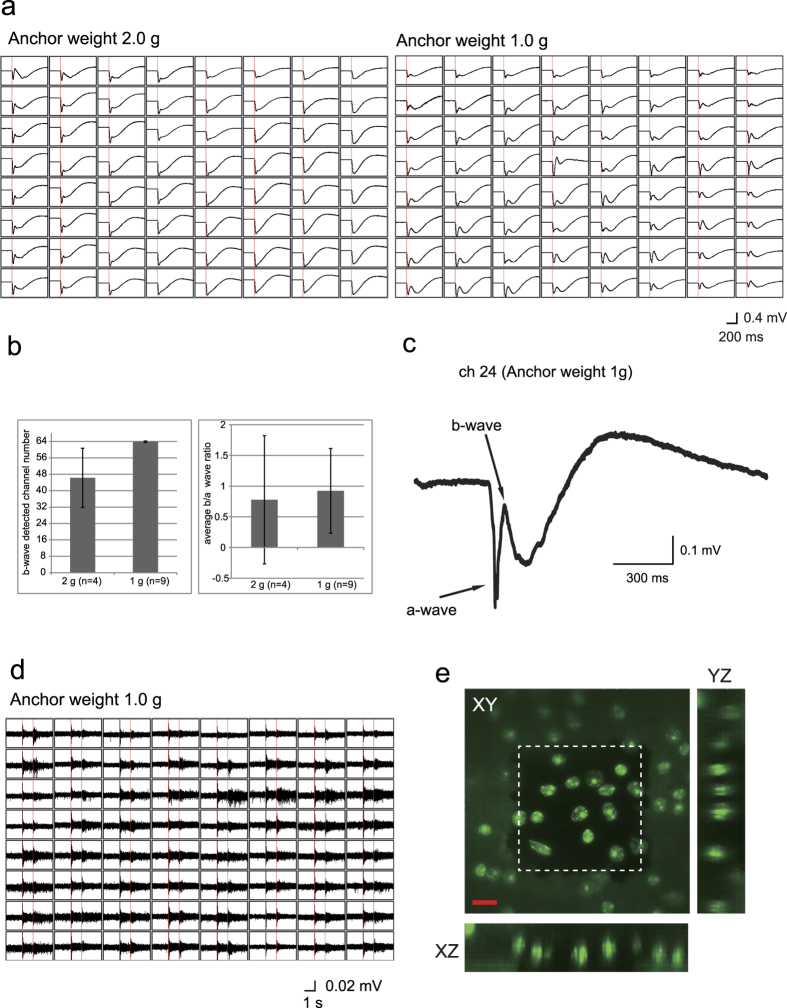
Representative micro ERGs (mERGs) and retinal ganglion cell (RGC) multiunit spike responses recorded with a multi electrode array (MEA) system from isolated C57BL/6 mouse retinas. (**a**) Averaged micro electroretinograms (mERGs) with 2 different anchor weights. The red line indicates the light stimulus. Eight x 8 panels of waves indicate the recordings from 64 electrodes. **(b**) The number of channels in which the b-waves were detected with the 1 g and 2 g anchor weights, and the averaged b/a amplitude ratios. “n” indicates the number of retinas tested. (**c**) Representative mERG from Channel 24 of Fig. 1a (averaged from the responses to 3 serial stimuli). (**d**) Multiunit RGCs responses in the retina in Fig. 1a with 1.0 g anchor (averaged from the responses to 3 serial stimuli). The red bar indicates the light stimulus onset and offset. (**e**) Representative RGC layer nuclei over one electrode after DAPI nuclear staining. The white dotted square indicated MEA electrode. The counted RGCs were of single layered structures in the XZ and YZ planes. Scale bar 10 μm.

Initially, we determined which weight of anchor will be better to record stable and repeatable mERGs (Fig. 1a). Because the MEA recording requires a good electrical contact of the retina to the electrodes, we tested anchors of 1 g and 2 g. Anchor weights of about 0.18 to 0.8 g are used in patch-clamp and brain slice imaging experiments, but we could not obtain stable recording with these light weight anchors. Instead, a 1 g anchor was better than 2 g weight in separating the a-and b-waves with stable b/a ratios, although the difference was not statistically significant between these 2 weights (Fig. 1a,b).

Representative mERGs, recorded with a 1 g anchor, are shown in Fig. 1c with a negative a-wave clearly separated from the following positive b-wave. In addition, we could record RGC spike responses with 1 s flashes with the settings optimized for mERGs (Fig. 1d). We confirmed that approximately 14 ± 6 cells were present in the GC layer over each electrode (50 μm × 50 μm square) by z-scan imaging with nuclei staining after the MEA recordings (Fig. 1e, n = 3 retina observed). This cell density is consistent with that reported^20^,^21^.

**Figure 2:**
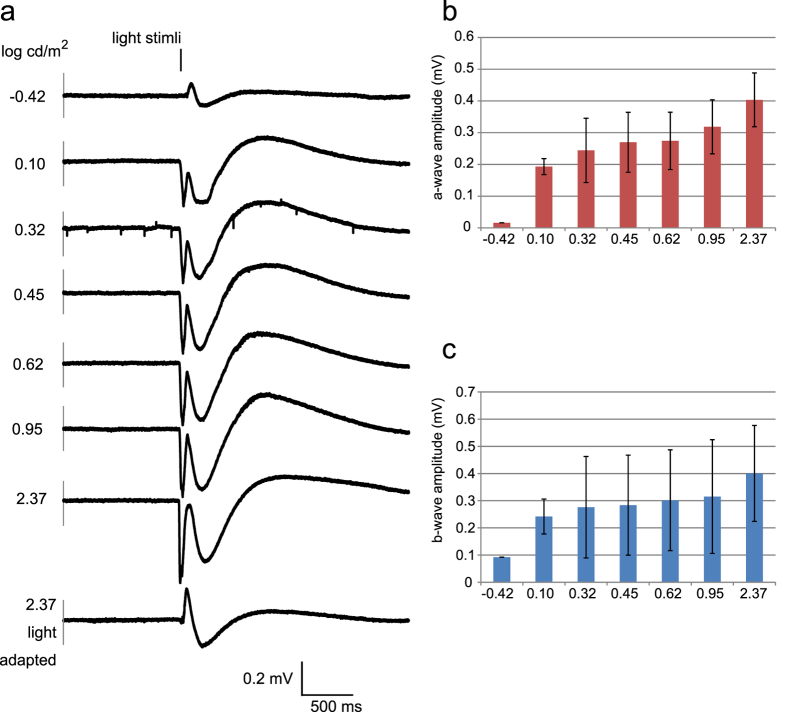
Light intensity dependency of mERGs of C57BL/6 mouse retinas. (**a**) Representative mERG responses elicited by different light intensities (averaged from the responses to 3 serial stimuli). (**b**) Averaged amplitude of a-waves from 64-channels of 3 retinas (mean ± SD). (**c**) Averages of amplitude of b-waves from 64-channels of 3 retinas (mean ± SD).

We evaluated the effect of the stimulus intensity on the amplitudes of the a- and b-waves of the mERGs in the dark-adapted retina. The results showed that the amplitudes of both the a- and the b-waves increased as the intensity increased, and the amplitudes of both waves decreased after 20 min of light-adaption by 1.67 log cd/m^2^ (Fig. 2 mean ± SD, 192 electrodes of 3 retinas).

### Pharmacologic analysis of components of mERGs

**Figure 3:**
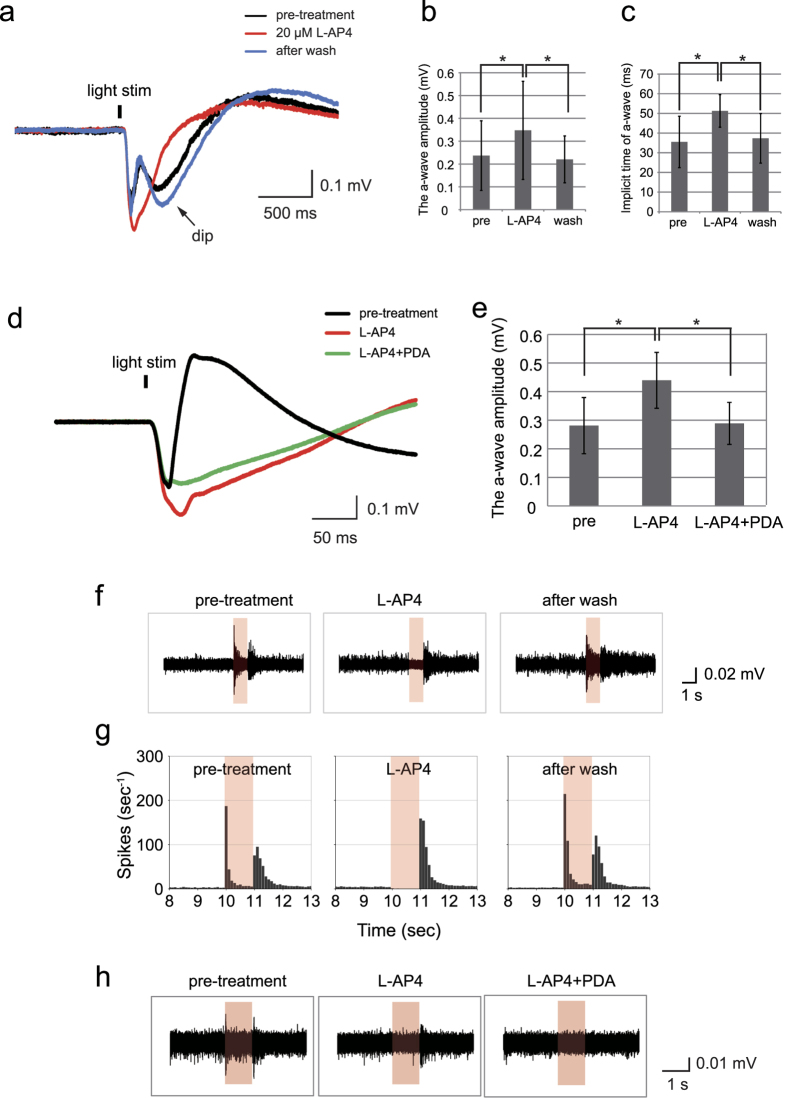
Pharmacological evaluations of mERG components and RGC spike responses in C57BL/6 mouse retinas. (**a**) Representative mERG patterns with and without exposure to 20 μM L-AP4 (average of 3 responses). (**b**) Effects of L-AP4 on the a-wave amplitudes (mean ± SD of 192 channels of 3 retinas). *P < 0.001, *Kruskal-Wallis test*. (**c**) Effects of L-AP4 on the implicit time of the a-wave (mean ± SD of 192 channels of 3 retinas). *P < 0.001, *Kruskal-Wallis test*. (**d**) Representative mERGs with or without exposure to L-AP4 and 6 mM PDA (averaged from the responses to 3 serial 0.45 log cd/m^2^ stimuli). Note that time calibration is modified in this figure for a better visualization of the changes in the a-wave. (**e**) Effects of L-AP4 only and L-AP4 with PDA on the a-wave amplitudes (mean ± SD of 192 channels in 3 retinas). *P < 0.001, *Kruskal-Wallis test*. (**f**) Representative RGC responses with and without L-AP4. (**g**) Corresponding peristimulus time histograms (PSTHs) of addition of spike numbers from all the 64 channels. All of the recorded responses were analyzed using spike sorting, and the summation of the firings of all of the RGCs are shown as PSTHs. (**h**) Representative RGC responses with L-AP4, and L-AP4 with PDA.

Next, we performed pharmacological experiments to determine the contributions of the different types of neurons to the a- and b-waves of the mERGs. When the isolated retina was exposed to 20 μM L-AP4, a group III mGluR agonist, the a-wave amplitude increased by about 46% (pre-treatment, 0.236 ± 0.152 mV; AP4, 0.347 ± 0.214 mV; after washout, 0.220 ± 0.102 mV; n = 3 retinas, p < 0.001, *Kruskal-Wallis test*), and the b-waves were completely abolished by the blocking of the ON bipolar cells (Fig. 3a,b). The implicit time of the a-wave was prolonged by 44% (pre-treatment, 35.5 ± 13.1 ms; AP4, 51.2 ± 8.3 ms; after washout; 37.3 ± 12.6 ms; p < 0.001, *Kruskal-Wallis test*; Fig. 3c). This result indicated that the activities of the bipolar cell components also contribute to the a-wave in mERG consistent with previous reports in monkeys^22^,^23^, cats^24^, and rabbits^25^. A small negative dip was seen following the b-wave before the L-AP4 treatment, and this dip was eliminated by exposure of the retina to L-AP4 (Fig. 3a). Exact cellular origins of this negative component after the b-wave were not determined, but we assumed that it can originate from third-order neurons receiving ON pathway input, such as negative scotopic threshold response (negative STR)^26^,^27^,^28^ or photopic negative response (PhNR)^29^,^30^.

To confirm that the mERGs was made up of neural activities of the inner retinal neurons, we also expose the retinas to *cis*-2,3-piperidine-decarboxylic acid (PDA), an antagonist of NMDA, AMPA, and the kainate types of glutamate receptors. PDA blocks the light driven responses of the OFF-bipolar cells, horizontal cells, many amacrine cells, and ganglion cells while the ON bipolar cells are relatively unaffected^23^,^31^. Exposure to PDA in addition to L-AP4 reduced the a-wave amplitude to 65.7% of that after exposure to L-AP4 alone (pre-treatment, 0.281 ± 0. 098 mV; L-AP4, 0.44 ± 0.097 mV; L-AP4 + PDA, 0.289 ± 0.073 mV; n = 3 retina; p < 0.001, *Kruskal-Wallis test*;Fig. 3d,e). The origin of these reduced components were supposed to be from the second- and third-order retina neurons of the OFF-pathway.

The results of these pharmacological studies indicated that the mERGs received contributions from the retinal cells that were similar to the full-field ERGs^32^,^33^,^34^,^35^,^36^,^37^.

### Effect of inhibition of glutamate receptors on RGCs responses

**Figure 4:**
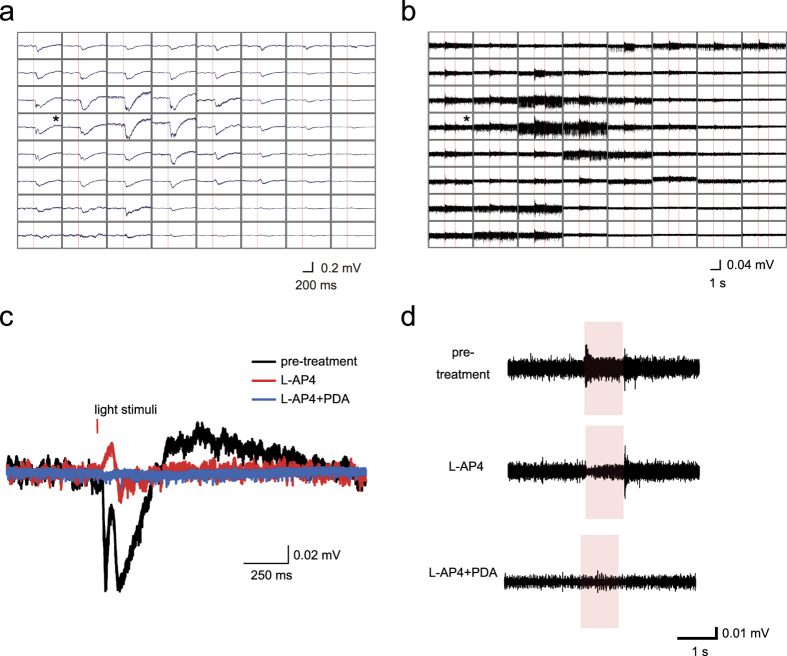
mERGs and RGC responses in *rd1-2J* mice. (**a,b**) Averaged mERGs and RGCs responses recorded by 64 channels from a 4-week-old *rd1-2J* isolated retina elicited by 3 serial stimuli of 0.45 log cd/m^2^. (**c**) Representative mERGs from the retina shown in Fig. 4a with L-AP4 and with additional PDA (recorded from the channel indicated by the asterisk in Fig. 4a). (**d**) Representative RGCs responses in the same channel as Figure 4c with L-AP4 and addition of PDA (recorded from the channel indicated by the asterisk in Fig. 4b).

In parallel with the mERG recordings, we recorded RGC spike responses before and after L-AP4 and PDA application (Fig. 3). Representative RGCs spikes elicited by 0.45 log cd/m^2^ full-field flashes (−1.54 log cd.s/m^2^) before and after the exposure to 20 μM L-AP4 are shown in Fig. 3f. The transient ON responses were abolished by L-AP4, and they recovered after a washout of L-AP4. This is consistent with the elimination and recovery of the b-waves of the mERGs. In the peristimulus time histograms (PSTHs) representing a summation of all of the RGC spikes from the 64 electrodes, the transient OFF responses were enhanced after the retina was exposed to L-AP4, and they returned to the pre-exposure level after a washout of L-AP4 (Fig. 3g). Additional exposure of the L-AP4 retinas to PDA abolished the OFF responses of the RGCs which is consistent with the elimination of the OFF components of the mERGs (Fig. 4g). These findings indicate that with the MEA recording system, we can obtain information on the RGCs activities from the areas corresponding to that of the mERGs which will confirm the interpretation of light-evoked intraretinal events.

### Detection of mERGs and RGCs responses in retinas with early photoreceptor degeneration

We then recorded mERGs from isolated retinas of the *rd1-2J* line of mice at 3- to 4- weeks, 5- to 6-weeks, and ≥7-weeks old *rd1-2J* mice elicited by 0.45 log cd/m^2^ stimuli. Representative mERGs and RGC spike responses from a peripheral part of the 4w *1-2J* retina are shown in Fig. 4a,b. Although the possibility of detecting mERGs and RGC spike responses with 0.45 log cd/m^2^ becomes increasingly low with increasing age (Table1), we could still detect reduced mERGs with the a- and the b-waves from the peripheral retinal area until postnatal 5-weeks-of-age.

We then performed pharmacological experiments in the degenerating retinas as was done in wild type retinas (n = 3). Surprisingly, exposure to L-AP4 abolished both the a- and b-waves in 2 retinas, and a small positive peak remained in one retina (Fig. 4c). Furthermore, exposure to L-AP4+PDA abolished the remaining responses completely in all of the retinas (n = 3). These findings indicate that the negative components in these progressive degenerating retinas originated not from the photoreceptors but mainly from second- or third-order neurons. The RGC spike responses from the same electrodes with Fig. 4c are shown in Fig. 4d. The L-AP4 treatment eliminated the ON responses and enhanced the OFF responses. Addition of PDA eliminated the RGCs spike responses.

**Figure 5:**
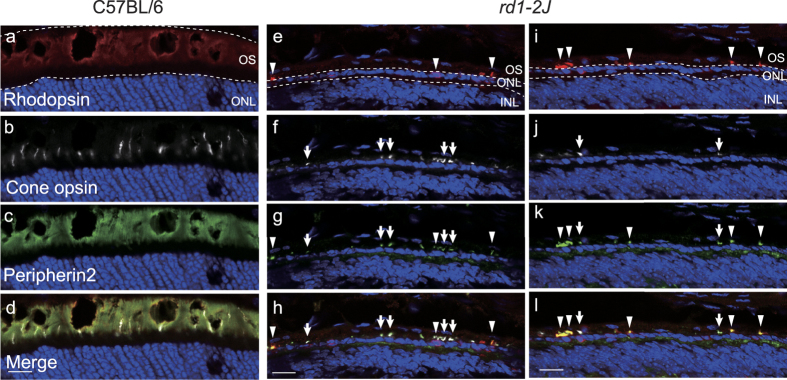
Expression of opsins and peripherin2 in C57BL/6 and 3 week old *rd1-2J* mice. The C57BL/6 mouse expressed rhodopsin (**a**), cone opsin (**b**) and peripherin2 (**c**). Three week old *rd1-2J* mouse had few rhodopsin positive cells (arrow heads) (**e**, **i**) and cones had short outer segments indicated by the colocalization of cone opsin (arrows) and peripherin 2 (**f**, **g**, **i**, **j**). In *rd1-2J* mice, 2 different fields are presented. Bars = 10 μm.

We also examined the histological appearances of the retinas of *rd1-2J* mice at 3- to 4-weeks-of-age by light microscopy (Fig. 5). There were few remaining rod photoreceptors but without outer segments (arrowhead) and a few cone photoreceptors with short outer segment-like structures (arrow). These structures stained positive for peripherin2 which are consistent with previous studies^38^.

### Classification of RGCs responses in degenerating *rd1-2J* mice retinas

Even with a minimal presence of photoreceptor contribution to the mERGs, there were light-responsive components in the mERGs of *rd1-2*J retinas for up to 5 postnatal weeks, and overall detection rate of RGC responses to light seemed higher than that of mERG during degeneration (Table 1). Therefore, we reviewed the changes in RGC responses from progressively degenerating retinas of *rd1-2J* by classification of the spike patterns of the ON responses.

**Figure 6:**
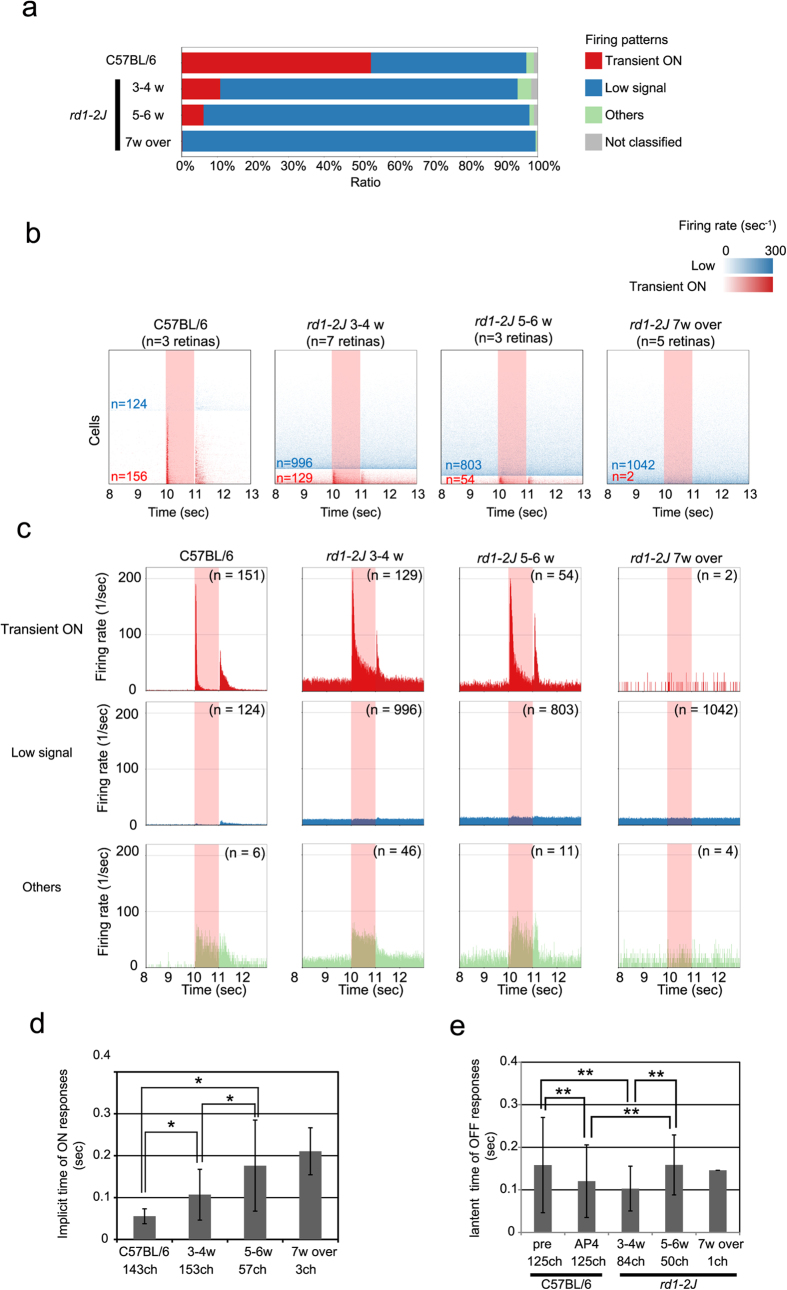
Classification of RGC responses in degenerating *rd1-2J* mice retinas. After spike sorting, all detected firing patterns were clustered into transient ON, sustained ON, delayed ON, and not-classified groups (see Methods for detail). (**a**) Distribution of firing patterns of individual cells in wild type (C57BL/6), and *rd1-2J* retinas of 3 age groups. Sustained and delayed ON are presented as “others”. (**b**) All of the spikes of the individual cells that were classified as transient ON (red) or Low signals (blue) are plotted along the horizontal time axis in raster plots. The colored numbers denote the number of cells clustered as transient ON (red) and low signal (blue). The pink shaded area in the middle of each panel indicates the light stimuli. Note that low signal group has overall lower signals than transient ON group. See Fig. 6c for quantified comparison. (**c**) Histograms presented as the summation of all firings of all the cells in each clustered firing pattern in wild type and *rd1-2J* of each age group. The numbers denote the cell number clustered in each group. (**d**) Implicit time to the transient ON peaks calculated on each channel with transient ON spikes are presented as the means ± SDs in the wild type retina and *rd1-2J* retinas of different age groups. Numbers indicate the number of channels with transient ON spikes. *P < 0.001, *Kruskal-Wallis test*. Seven-week old sample was excluded from the statistic analysis because of the small number of positive channels. (**e**) Implicit time to the transient OFF peaks calculated on each channel with transient OFF spikes are presented as the means ± SDs in the wild type retina and *rd1-2J* retinas of different age groups. Numbers indicate the number of channels with transient ON spikes. **P < 0.005, *Kruskal-Wallis test*. Seven-week old sample was excluded from the statistic analysis because of the small number of positive channels.

After spike sorting, the spike patterns in response to “ON” signals from individual sources, or “cells”, were classified into transient ON, low signals, and other signals including sustained/delayed-ON signals (Fig. 6a). The majority of the spikes fell into the transient ON or low signal categories with the light intensity used. The spike patterns of individual cells in transient ON and low signal categories were then plotted separately along the horizontal time axis in red and blue raster plots, respectively, for better visualization of all the analyzed retinas of wild type and *rd1-2J* of each age group (Fig. 6b).

In the wild type retinas, 53% of the cells had transient ON responses but in *rd1-2J* retinas, approximately 10% of the cells had transient ON responses at 3- to 4-weeks-of-age, 6% at 5- to 6-weeks-of-age, and there were hardly any cells with transient ON responses at 7 weeks or older. The averaged spiking pattern of each category in each age animal group is presented as PSTHs (Fig. 6c). Low signal category included the cells with no definite ON responses, and some with OFF responses instead, and others had spontaneous spiking activity.

We also studied the implicit times of the transient ON peaks in the same age groups (Fig. 6d). The implicit times of the transient ON peaks were significantly longer in 3- to 4-week-old *rd1-2J* mice than wild type mice (p < 0.001, *Kruskal-Wallis test*), and the remaining transient ON spike peaks were more delayed with progression of the disease. On the other hand, the implicit times of the transient OFF response were significantly shorter in 3- to 4-week-old *rd1-2J* mice (p < 0.005, *Kruskal-Wallis test*) similar to those in L-AP4 treated wild type retinas (Fig. 6e).

## Discussion

We previously showed that the MEA system can record focal mERGs with good repeatability which then allowed us to detect neural activity of each electrode in a point-by-point fashion^17^. We have refined our protocol to study focal retinal function in a more detailed manner by identifying the neural elements contributing to the a- and the b- waves by using two of glutamate receptor inhibitors. We also found a light intensity dependency of the a- and b- waves, and we were able to apply this system to mice with progressive photoreceptor degeneration.

In order to obtain a good mERG, it was necessary to maintain an adequate electrical contact between the retina and the electrodes. Depending on the types of mesh material, size, or density, a heavier anchor was considered to damage the retinal tissue. Thus, appropriate conditions including the anchor weight must be determined to record reliable responses. With our system, using mice retinas, 1 g weight anchor was better than a 2 g anchor to record the a- and b- waves. The use of a perforated MEA may also help to minimize these problems. Also, the use of the optimized conditions for recording the mERG responses also worked well for RGC spike recordings.

In the pharmacological analysis, L-AP4 application clearly eliminated the b-waves and enhanced the a-wave amplitude, indicating that the inhibition of the ON bipolar pathway led to these changes. These changes were reversed after washing out the L-AP4. A small negative dip following the b-wave was also eliminated by application of L-AP4. The exact origin of this negative dip is unknown, but can be ON pathway activities from the inner retina including negative STR and PhNR^26^,^27^,^28^.

Then, an exposure to PDA in addition to AP-4 almost reversed the increased a-wave amplitude by AP-4, possibly due to the elimination of the negative components contributed by the OFF bipolar cells and third-order retinal neurons. The remaining negative component after AP-4 and PDA application was considered to be of photoreceptor origin. These results indicate that the mERGs consisted relatively similar components as regular corneally recorded full-field ERGs. The results of the RGC responses and mERGs were quite consistent, and support each other in the interpretation of the intraretinal events.

Next, we extended the use of MEA by determining whether the mERGs and RGC spike responses could be recorded from *rd1* mice. Mice of the *rd1* lines are one of the most commonly used retinal degeneration models, and they have been used to determine strategies for treating human retinal diseases including protective interventions and regenerative studies for transplantation. The *rd1* mice are a group of mice strains and lines with a phenotype of very progressive photoreceptor degeneration. Histological studies of *rd1* mice showed that the rods were completely degenerated in the first 3 to 4 weeks of life^10^,^19^,^39^,^40^. We confirmed that this was also the case with our *rd1-2J* line. It has also been shown that full-field ERGs could not be recorded at any age from *rd1* mice^41^,^42^,^43^, and therefore it was not known if these mice had any visual function during development. Our studies showed that distinct a- or b-waves as well as transient ON and OFF RGC multiunit responses could be recorded in the peripheral part of the *rd1-2J* retinas at 3- to 6-weeks-of-age. Surprisingly, however, L-AP4 application almost eliminated both the a- and b-waves, and the effect was complete with additional PDA. The effect with this dose of L-AP4 was reversible, and the corresponding RGC shift from transient ON to transient OFF pattern excluded the possibility of drug toxicity. The results indicate that, even though the contribution of the photoreceptors to the a-wave is minimal, the a- and/or b-wave-like responses can still be elicited from this degenerating retina. The major contribution of third order neurons to negative components in ERGs has been reported in other models of photoreceptor degeneration including RCS rats or sodium iodate-treated rats^38^,^44^ which may also explain our observation.

Even with the absence of photoreceptor-driven components, the transient ON responses were recorded in 10% and 6% of all the RGCs with detected spikes including all the spontaneous and background spikes in *rd1-2J* retinas of 3–4-weeks and 5–6-weeks-of-age, respectively. Implicit time to transient ON responses also became prolonged with degeneration. All of these results suggest that the retinal visual circuit may have developed to some extent in some parts of the retina in *rd1* mice, but may be rapidly lost with increasing age. This may justify the use of this model for studies whose aim is to ‘restore’ and further rebuild visual function after some kind of repair process.

Some of the drawbacks of this study of the mERGs the first of which was the variability in the waveform of mERGs within the same retina which might be caused by uneven attachment of the retina to the individual electrodes, focal damages to the outer segments in the preparation procedures, varying degrees of remaining RPE on isolated retinas and/or status of dark-adaptation after repeated light stimuli. Additionally, although our mERG recordings stably isolated the b-waves, the b-wave amplitudes were sometimes much higher in some series of experiments compared to others (Fig. 3D). By using some other methods of *ex vivo* ERG recording, b-waves were reportedly smaller than those observed *in vivo* ERGs without an addition of divalent metal ions such as Ba^2+^, Zi^2+^ and Ni^2+^ 45,46,47. They reported the increase in b-waves by adding BaCl_2_ that inhibited glial slow PIII component, and by adding NiCl_2_ or ZnCl_2_ that inhibited GABA release from horizontal or amacrine cells. It would also be useful to add these ions to reduce b-wave variability in mERG recordings using MEA.

The second was that we were able to analyze only a small area of 1.05 mm × 1.05 mm with the present system. Still, detailed analyses of the specific area of interest would help evaluate the status of small areas such as a grafted area in regenerative studies, which is otherwise very difficult. In addition and most importantly, by characterizing the focal retinal function as being not just an “all or none” type analysis, this 2-way approach using mERG combined with RGC spike recordings may also be helpful in interpreting the intraretinal connection such as host-graft interaction in evaluating if the graft light response is transmitted to host RGCs.

In conclusion, we showed that mERGs and RGC multiunit spike responses can be recorded with the MEA system from isolated mouse retinas. The responses were reliable and had similar components of the *in vivo* full-field ERG responses. Our results showed that this system can be used to analyze the retinas during the degenerating process and hopefully during the regenerating process.

## Methods

All animal experiments were conducted in accordance with local guidelines and the ARVO statement on the use of animals in ophthalmic and vision research. All of the experimental protocols were approved by the committee of the RIKEN Center for Developmental Biology (CDB).

### Animals

Adult C57BL/6N mice were obtained from the RIKEN CDB animal experiment committee. The C57BL/6J-*Pde6b*^*rd1-2J*^/J (*rd1-2J;* same as *nmf137*) mice were obtained from the Jackson Laboratory (Bar Harbor, ME, USA). The C3H/He, C3H/HeJ, and C3H/HeN mice were obtained from Nihon SLC (Shizuoka, Japan) and used for building templates for RGC spike pattern clustering (see “Generating templates of light responses of RGCs” in METHODS). The animals were maintained under a 12-hour light-dark cycle, and they were dark-adapted overnight before the eyes were used for the MEA experiments.

### Isolation of retina

Neural retinas were prepared for recordings as described in detail with minor modifications^17^. Briefly, rodents were anesthetized with sevoflurane (Abbott Japan, Osaka, Japan) and sacrificed. The eyes were enucleated and the neural retinas were isolated from the RPE and sclera and vitreous under dim red light. After trimming 2 to 3 mm off of the margins of the retina, the retina was mounted over a MED64 probe with the ganglion cell against the electrodes. The MED64 probe consisted of 64 electrodes (50 μm × 50 μm square, interpolate distance 150 μm, MED-P5155, Alphamed Scientific Inc., Osaka, Japan). The MED probes were treated with 0.1% polyethyleneimine (Sigma-Aldrich, St Louis, MO, USA) in 25 mM borate buffer (pH 8.4) over night at room temperature.

Custom-made anchors were made of stainless steel washers (weighing 1.0 or 2.0 g), with nylon and polyurethane mesh attached with glue for every experiment. Then, the mounted retina was placed in a chamber and continuously perfused with bicarbonate-based Ames’ medium (Sigma-Aldrich, St Louis, MO, USA) gassed with 95% O_2_ and 5% CO_2_ maintained at 34–37 °C with a temperature controller (TC324B, Warner Instruments, Hamden, CT).

### Recordings with multi electrode array (MEA) system

After 30 min of recovery, mERGs and multiunit responses from the RGCs were recorded with the MED64 system (Alphamed Scientific Inc., Osaka, Japan). The signal were amplified and filtered between 1 Hz and 10 kHz for the mERGs and 100 Hz and 10 kHz for the RGC multiunit spike responses. The RGC multiunit signals were also filtered before the spike sorting.

The light stimuli were obtained from white LEDs (NSPW500C, Nichia Corp., Tokushima, Japan) and their luminance, duration, and frequency were controlled by an electronic pulse generator (DSP-420, Dia-Medical System, Tokyo, Japan). A stimulus duration of 10 ms was used to elicit the mERGs and 1 s stimuli were used to elicit the RGC spike responses. In our standard protocol, a three-time serial stimuli were repeated with an interval of 1 min, and a single recording time was 10 s for the mERGs and 21 s for the RGC multiunit recordings.

The light intensities were measured with an optometer (ModelS471 and 268R, Gamma Scientific, CA, USA). The regularly used light intensity was 0.45 log cd/m^2^ which is equivalent to 0.0285 (−1.545 log) cd.s/m^2^ for the 10 ms mERG stimulus.

In the pharmacological studies, L-2-amino-4-phosphonobutyric acid (L-AP4; Wako, Osaka, Japan) was used at a concentration of 20 μM, and *cis*-2,3-piperidine-dicarboxylic acid (PDA) was used at 6 mM. In the *rd1* experiments, 20 μM of AA92593 (Sigma-Aldrich, St Louis, MO, USA) was added to the Ames’ medium to block the activity of the intrinsic melanopsin ganglion cells.

### Nuclear staining and immunohistochemistry

For nuclear staining of wholemount retinas, neural retinas were fixed after the MEA recordings with 4% paraformaldehyde in phosphate buffered saline (PBS) at room temperature for 30 min on the MED64 probe. Then the specimens were washed 3 times with PBS and stained with 4′,6-diamidino-2-phenylindole (DAPI, Thermo Fisher Scientific, USA, MA) in 0.05% Tween-20 (Nakalai Tasque, Japan, Kyoto)/PBS at room temperature for 20 min, and wash with PBS 3 times. Then the tissue was cleared with 60% 2,2′-thiodiethanol (TDE) in PBS and z-stack images were obtained with a confocal microscope (SP8, Leica microsystems, Welzlar, Germany) from randomly selected 10 electrodes from 3 retinas, and the number of nuclei in the RGC layer were counted and averaged.

For immunohistochemistry, the retinas from 3-week-old *rd1-2J* mice were fixed with 4% paraformaldehyde in PBS at room temperature for 30 min and with 30% sucrose in PBS at 4 °C overnight. Then the eyes were embedded in OCT compound (Tissue Tec O.C.T. compound, Sakura Finetek Japan Co. Ltd., Tokyo, Japan), frozen at −30 °C and sectioned at 10 μm with a cryostat (HM560, Thermo Fischer Scientific, MA, USA). Then, the sections were stained with primary antibodies: mouse anti-rhodopsin at 1:1000 (clone Ret P1, Sigma-Aldrich, St Louis, MO, USA); goat anti-OPN1SW at 1:200 (Santa Cruz Biotechnology, Inc., TX, USA); and rabbit anti-peripherin2 at 1:500 (Proteintech, IL, USA). The secondary antibodies were anti-mouse/goat/rabbit IgG conjugated with AlexaFluor546/647/488 at 1:1000 (Thermo Fischer Scientific, MA, USA) and DAPI at 1:1000 in 1% donkey serum. The stained sections were mounted in FluoSave reagent (Merch Millipore, Darmstadt, Germany) and were examined with a confocal microscope (SP8, Leica microsystems, Welzlar, Germany).

### Spike sorting

The RGC multiunit responses recorded by the MED64 system were imported into the Spike2 software (version 7.11c; Cambridge Electronic Design, Cambridge, UK) by the implemented converting function. Each recording had 64 channels of RGC multiunit responses that was recorded from the isolated retina, and each channel was filtered by a second order Butterworth band pass filter (200 to 2800 Hz) followed by spike sorting. The signals from each channel were separated into individual units representing the spike responses from individual RGCs. The time series of individual spikes were exported into text files for further analysis. The implicit times of the transient ON and transient OFF responses were manually calculated after spike sorting using the peristimulus time histograms (PSTHs) for each channel (bin size 0.1 sec).

### Generating templates of light responses of RGCs

To obtain a typical neural activity pattern during a light response, we used the time-series of individual spiking data of C57BL/6N mice (n = 3), of younger and older *rd1-2J* mice (3 to 36 weeks, n = 15), and other *rd1* mouse lines (3w C3H/HeJ; n = 4, 3w C3H/HeN; n = 6). R (version 3.2.1, R Core Team, Vienna, Austria)^48^ and an originally written script was used for the analysis. First, the spiking data were converted to firing frequency data with a bin size of 100 ms. A three second window beginning two seconds before the light stimuli and ending at the end of the light stimuli was used to characterize the responses. Three thousand out of 13609 light responses were randomly selected, but 1008 out of 3000 light responses were not analyzed because there were no spikes during the time of the window of interest. The remaining 1992 light responses were annotated as any of ‘transient ON’, ‘delayed ON’, ‘sustained ON’ or ‘low signal’ by visual inspection. These patterns were used as a training dataset for classification of the light responses recorded in this study.

### Classification of light responses by deep learning

The deep learning model for classification of light responses was built on an open source machine learning platform H2O (version 3.6.0.8, H2O.ai, CA, U.S.A.)^49^. The manually classified light response dataset was used for training and validating the model. Once the model was optimized, further classification of the light responses was done by the prediction based on this model. A single spike-sorted cell has three serial light responses. When more than two responses out of three were classified into an identical group, the cell was classified to the group (‘transient ON’, ‘low signal’, ‘sustained ON’, or ‘delayed ON’). When all three responses were classified into different groups, the cell was classified as “not classified (N.C.)”. See Supplementary Document for the classification source codes (https://goo.gl/lzMfY2 ). Two source codes are included in this document. “train.R” is the source code for machine learning which build the decision model. “predict.R” is the source code for prediction applying the trained model.

## Additional Information

**How to cite this article**: Fujii, M. *et al*. Evaluation of micro Electroretinograms Recorded with Multiple Electrode Array to Assess Focal Retinal Function. *Sci. Rep.* **6**, 30719; doi: 10.1038/srep30719 (2016).

## Figures and Tables

**Table 1:** Response ratio to light stimulus of 0.45 log cd/m^2^ in *rd1-2J* mice retinas of different age groups.

		3-4w	5-6w	7w-
mERG		4/7 (94/448ch)	1/3 (33/192ch)	0/5 (0/320ch)
RGC response	ON	6/7 (153/448ch)	2/3 (57/192ch)	1/5 (3/320ch)
	OFF	6/7 (84/448ch)	2/3 (50/192ch)	1/5 (1/320ch)

Each strain was evaluated for the presence of mERGs and transient ON or OFF RGC multiunit responses elicited by 0.45 log cd/m^2^ stimuli at indicated postnatal age in weeks. The response ratios are the number of samples with responses/total number of retinas tested. Also, the number of channels with detected responses of all types/64 channels x tested retinas.
